# The Relationship between Early Childhood Blood Lead Levels and Performance on End-of-Grade Tests

**DOI:** 10.1289/ehp.9994

**Published:** 2007-04-27

**Authors:** Marie Lynn Miranda, Dohyeong Kim, M. Alicia Overstreet Galeano, Christopher J. Paul, Andrew P. Hull, S. Philip Morgan

**Affiliations:** 1 Nicholas School of the Environment and Earth Sciences, Duke University, Durham, North Carolina, USA; 2 Sociology Department, Duke University, Durham, North Carolina, USA

**Keywords:** disparities, lead levels, school performance

## Abstract

**Background:**

Childhood lead poisoning remains a critical environmental health concern. Low-level lead exposure has been linked to decreased performance on standardized IQ tests for school-aged children.

**Objective:**

In this study we sought to determine whether blood lead levels in early childhood are related to educational achievement in early elementary school as measured by performance on end-of-grade (EOG) testing.

**Methods:**

Educational testing data for 4th-grade students from the 2000–2004 North Carolina Education Research Data Center were linked to blood lead surveillance data for seven counties in North Carolina and then analyzed using exploratory and multivariate statistical methods.

**Results:**

The discernible impact of blood lead levels on EOG testing is demonstrated for early childhood blood lead levels as low as 2 μg/dL. A blood lead level of 5 μg/dL is associated with a decline in EOG reading (and mathematics) scores that is roughly equal to 15% (14%) of the interquartile range, and this impact is very significant in comparison with the effects of covariates typically considered profoundly influential on educational outcomes. Early childhood lead exposures appear to have more impact on performance on the reading than on the mathematics portions of the tests.

**Conclusions:**

Our emphasis on population-level analyses of children who are roughly the same age linked to previous (rather than contemporaneous) blood lead levels using achievement (rather than aptitude) outcome complements the important work in this area by previous researchers. Our results suggest that the relationship between blood lead levels and cognitive outcomes are robust across outcome measures and at low levels of lead exposure.

Although much progress has been made, childhood lead poisoning remains a critical environmental health concern. Since the late 1970s, mounting research demonstrates that lead causes irreversible, asymptomatic effects far below levels previously considered safe. Thus, the Centers for Disease Control and Prevention (CDC) lowered incrementally its intervention threshold for lead levels considered dangerous in children by 88% from 60 to 10 μg/dL over the last 40 years (CDC 2006). The 2003–2004 National Health and Nutrition Examination Survey (NHANES) survey data reveal blood lead levels at or above the CDC blood lead action level of 10 μg/dL in 2.3% of 1- to 5-year-olds in the United States, with children tested having an overall geometric mean blood lead level of 2.1 μg/dL (National Center for Health Statistics 2006). These data indicate that > 500,000 children < 6 years of age currently experience blood lead levels at or above the CDC blood lead action level of 10 μg/dL (U.S. Census Bureau 2002).

Low-level lead exposure, including prena-tal exposure, has been linked to decreased performance on standardized IQ tests for school-age children (Bellinger et al. 1992; Canfield et al. 2003; Chiodo et al. 2004; Dietrich et al. 1993; Schnaas et al. 2006; Tong et al. 1996). A meta-analysis conducted by Schwartz (1994) estimated that a 10-μg/dL increase in blood lead causes a 2.6-point decrease in IQ level. Dudek and Merecz (1997) observed a statistically significant relationship between blood lead and IQ in a population of 380 children with an average blood lead level of 10.2 μg/dL. The analysis finds that the most severe declines occur in children with blood lead levels between 5 and 10 μg/dL. Not only is there a correlation between blood lead levels and a decrease in IQ, but the slope of the IQ–lead regression is steeper at the lowest levels (Lanphear et al. 2005; Needleman and Landrigan 2004; Schnaas et al. 2006; Schwartz 1993). Needleman and Landrigan (2004) state that this indicates that significant damage occurs at the lowest levels of exposure.

Another study examining repeated blood lead levels in children followed from < 1 to 5 years of age detected steeper declines in cognitive abilities in children whose maximum blood lead level never reached 10 μg/dL (Canfield et al. 2003). Linear modeling incorporating the full range of data indicates a 0.46-point decrease in IQ for every 1-μg/dL rise in blood lead level (Canfield et al. 2003). However, linear modeling restricted to blood lead levels < 10 μg/dL indicates a 1.37-point decrease in IQ for every 1-μg/dL rise in blood lead level (Canfield et al. 2003). Nonlinear modeling indicated a 7.4-point decrease in IQ as lifetime average blood lead levels rise from 1 to 10 μg/dL and a 2.5-point decrease in IQ as lifetime average blood lead levels rise from 10 μg/dL to 30 μg/dL (Canfield et al. 2003). Although the shifts in IQ are relatively small, the shifts are both important on a population scale and could be an indicator for other adverse neurologic effects in the individual (Rogan and Ware 2003).

Thus, research suggests that significant adverse health effects occur at blood lead levels below the current CDC blood lead action level, leading several researchers to call for its lowering. Learning and behavioral deficits may occur at blood lead levels < 5 μg/dL (Canfield et al. 2003; Chiodo et al. 2004; Lanphear et al. 2000; Schnaas et al. 2006). Meta-analysis and reviews suggest that any level of exposure is potentially detrimental (Gatsonis and Needleman 1992; Lanphear et al. 2005; Schwartz 1993, 1994). In a recent review article, Gilbert and Weiss (2006) called for reducing the CDC blood lead action level to 2 μg/dL.

Linking blood lead surveillance data with end-of-grade testing data for several counties in North Carolina, this study explores the potential relationship between early childhood lead exposure and educational achievement in elementary school. The objective of the current study is to determine whether blood lead levels in early childhood are related to educational achievement in early elementary school as measured by performance on end-of-grade testing. In undertaking this study, we link two large databases generated by two different offices of the State of North Carolina in the same populations but at different time periods.

## Methods

### Study area

**Figure 1:**
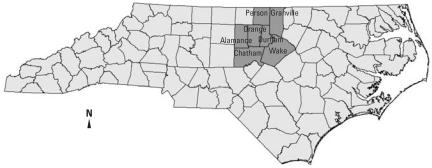
Study counties in North Carolina.

Our study focuses on seven counties in the Piedmont region of North Carolina (Figure 1). By assessing adjacent counties jointly, we account in part for migration patterns across counties in North Carolina and thus capture more children in the linking process.

### Data

Key data for this study include blood lead surveillance data from the state registry maintained by the North Carolina Childhood Lead Poisoning Prevention Program of the Children’s Environmental Health Branch, North Carolina Department of Environment and Natural Resources in Raleigh, North Carolina (2004), and educational testing data from the North Carolina Education Research Data Center (NCERDC) of Duke University, in Durham, North Carolina (2006). Methods for receiving, storing, linking, analyzing, and presenting results related to this study were all governed by a research protocol approved by the Duke University Institutional Review Board.

The blood lead surveillance data include child name, birth date, test date, blood lead level, type of test (venous or capillary), and home address. The North Carolina State Laboratory for Public Health (Raleigh, NC) conducted 90% of the lead analyses of the blood samples. The limit of detection for lead in blood as analyzed by the State Laboratory is 1 μg/dL, but all children whose blood lead levels are below the level of detection are assigned a value of 1 μg/dL in the state database. Blood lead levels are stored in the state database as integer values only. Most of the samples were sent to the State Laboratory from private providers, indicating that the samples were collected by trained health care professionals. Thus we can be confident in the consistency of blood lead sample collection across samples. We used blood lead screening data from 1995–1998. During this period, North Carolina estimates that it screened between 21.9 and 30.4 percent of children 1 and 2 years of age (North Carolina Childhood Lead Poisoning Prevention Program 2004). In theory, all children whose parents responded “yes” or “don’t know” to any of the three questions on the CDC Lead Risk Assessment Questionnaire (CDC 1997) should have been screened for lead, but it is difficult to ascertain true practice at the time.

Children in grades 3–8 are tested in reading and mathematics in North Carolina at the end of the school year. These assessments are “curriculum-based multiple-choice achievement tests…specifically aligned to the *North Carolina Standard Course of Study”* (North Carolina Public Schools 2004). The Reading End of Grade (EOG) test consists of multiple choice questions that cover *a*) cognition, *b*) interpretation, *c*) critical stance, and *d*) connections (North Carolina Public Schools Accountability Services Division 2006). The Mathematics EOG consists of multiple choice questions that cover *a*) number sense, numeration, and numerical operations; *b*) spatial sense, measurement, and geometry; *c*) patterns, relationships, and functions; and *d*) data, probability, and statistics (North Carolina Public Schools 2004).

The NCERDC maintains a database with records of all EOG test results statewide for tests from the 1995–1996 school year to the present (North Carolina Education Research Data Center 2006). This database includes identifying information such as name and birth date. Additionally, the database contains data on demographics and socioeconomics, testing conditions such as modifications, computer use, English proficiency, and school district. These data can also be linked longitudinally for all years each child has taken EOG tests in North Carolina.

Children who were screened for lead between the ages of 0 and 5 years from 1995 through 1998 in seven study counties (36,070 records for 35,815 children) were linked to their records in the 4th-grade EOG testing data in age-corresponding years. The early childhood environmental data (blood lead levels) were linked to elementary school educational outcome data (EOG test results) using 16 different combinations of social security number, date of birth, county federal information processing standards code, and first and last name. The linking schemas were designed to ensure accuracy while trying to achieve the highest number of linked records possible. Records that were linked were given a code for the particular type of linking method used, which enabled each method to be reviewed for the number of accurate matches that it provided. Each of the linking methods used educational data from 2000 to 2004, which allowed individuals to potentially be linked from the blood lead surveillance data to multiple end-of-grade tests from the educational data. Our process linked 42.2% of screened children to at least one EOG record. The percent linked for each county ranges from 24.4% for Orange County to 44.9% for Alamance County.

Assessing educational achievement based on standardized testing data is especially problematic for children for whom English is a second language. Thus we restricted our analysis to students who self-reported race as either white or black and who did not report any limited English proficiency. In so doing, we decreased our linked sample size by roughly 8%. We conducted all analyses on 4th-grade scores, both reading and mathematics. The final linked data set for 4th-grade reading and mathematics results contained 8,603 and 8,627 observations, respectively. Table 1 provides average blood lead levels for subgroups within the final linked data sets. As expected, migration or movement among these counties is significant, and roughly 6.7% of children were tested for blood lead levels in one county but sat for their end-of-grade testing in another county.

We employed both descriptive and multivariate statistical methods in our analysis, including Mantel-Haenzel chi-square tests to check equality of distributions of the black and white subsamples, and three different multivariate models to regress the EOG scores on a series of covariates. All models controlled for the following covariates as listed in the EOG test data: sex and race as standard demographic variables; participation in the free or reduced-price lunch program as a measure of socioeconomic status; parental education as a proxy for parental IQ and as a measure of socioeconomic status; daily computer use as a measure of stimulation in the home environment; and whether the school is a charter school, which in North Carolina is typically a measure of lower socioeconomic status of the enrolled children as a group. We included a covariate for age at which the blood lead screen occurred (taken from the blood lead screening data) to control for age-dependent effects of lead exposure. We also incorporated dummy variables for each of the school systems. The three models differed only by how the blood lead level variables are constructed in the model—as a continuous variable or multiple dummy variables. The models are compared via several test statistics such as adjusted *R*^2^, Akaike Information Criterion (AIC), and root mean squared error (MSE). All analyses were conducted using STATA 9.2 (StataCorp., College Station, TX).

## Results

We began our descriptive analysis by examining patterns in the linked data. For space reasons, we present here only the descriptive statistics for 4th-grade reading results. The 4th-grade mathematics results follow strikingly similar patterns. The multivariate analyses presented below include both 4th-grade reading and mathematics.

**Figure 2:**
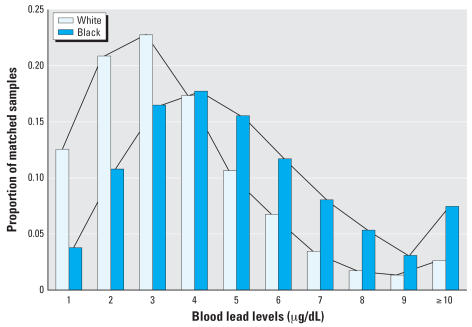
Distribution of blood lead levels among white and black children.

Figure 2 shows the distribution of children across blood lead levels and race categories. Of the total linked children for 4th-grade reading, 44.8% are white and 55.2% are black. Compared with black children, white children are overrepresented in the lower blood lead level categories (blood lead level, 1 to 3) and underrepresented in the higher blood lead level categories (blood lead level, 4 to ≥ 10). This blood lead level cut point at 3 holds for the 4th-grade mathematics scores as well.

Figure 2 thus demonstrates a distribution for black children that is shifted to the right and is characterized by higher variance compared with white children. These sample distributions are statistically different from each other. Construction of a dissimilarity index indicates that 25% of the members of one group would need to be reassigned blood lead levels for the two groups to show equivalent blood lead level distributions. The Mantel-Haenszel chi-square test for equality of distribution shows the two sample distributions to be statistically significantly different from each other (*p* < 0.0001).

**Figure 3:**
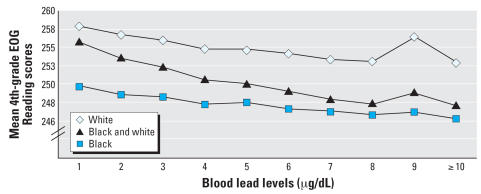
4th-grade mean Reading EOG test results stratified by blood lead levels.

Figure 3 shows the mean reading scores by race and blood lead levels for all linked students in the 4th-grade reading data set, disaggregated by race. This graphic shows a clear negative relationship between test scores and blood lead levels: Higher blood lead levels are associated with lower test scores, with some erratic behavior at blood lead levels of 9 μg/dL, likely due to the small sample size at this higher blood lead level.

**Figure 4:**
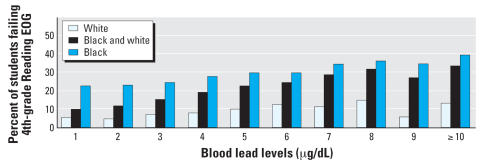
Percent of students failing 4th-grade Reading EOG.

At the lower end of the achievement scale, Figure 4 also demonstrates a dose–response effect between blood lead levels and failure on the end-of-grade test. Subgroups of children with lower blood lead levels in early childhood have lower failure rates on both the mathematics and reading end-of-grade tests (data shown only for 4th-grade reading data set); subgroups with higher blood lead levels in early childhood have higher failure rates.

Although this descriptive evidence is consistent with claims of a causal relationship between blood lead levels and test performance, alternative interpretations are plausible and can be addressed using multivariate analysis. For instance, given the higher blood lead level for children of lower socioeconomic status (as measured by free/reduced-price lunch and low parental education), perhaps these factors are responsible for the observed association of blood lead levels and test scores. Thus we used multivariate analysis to control for the covariates noted in “Methods.” The referent group is defined as white female students, enrolled in the Wake County School System, who do not participate in the free or reduced-price lunch program, who do not use a computer daily, and whose parents graduated high school.

To explore the functional form of the association between the lead variable and test scores, we compare three alternative specifications. The 6 analyses (3 models × 2 data sets) are presented in Tables 2 and 3.

In all models, the coefficients on the covariates are of the expected sign. The coefficient on the age at which the blood screen occurred is negative and highly significant, indicating that a higher blood lead level at a later age has a stronger depressive effect on test performance. This likely results from the fact that children who have high blood lead levels at 4 or 5 years of age typically would have had even higher blood lead levels at 2 or 3 years of age, given that the latter is typically considered the age of peak exposure (Canfield et al. 2003; CDC 1997; Dietrich et al. 2001).

The first model represents blood lead level as a continuous variable: We constrain the effect of a one-unit increase in blood lead level to be identical over the full range of observed scores. The coefficient on blood lead level is negative and statistically significant for 4th-grade reading and 4th-grade mathematics (both *p* < 0.0001). This effect and others discussed below are net of all control variables shown in the table.

The second model includes two dummy variables: one that is set equal to 1 if the blood lead level is 5–9 μg/dL; and one that is set equal to 1 if the blood lead level is ≥ 10 μg/dL. The coefficient on the dummy variable for a blood lead level of 5–9 μg/dL is negative and significant in both the reading and mathematics models (both *p* < 0.0001). In addition, the coefficient on the dummy variable for a blood lead level of 10 μg/dL is negative and significant in both the reading and mathematics models (again, *p* < 0.0001). In analysis not shown here, we also estimated a model that used a single dummy variable for blood lead level ≥ 5 μg/dL and a separate model with a single dummy variable for blood lead level ≥ 10 μg/dL. The results in Tables 2 and 3, in comparison with other models not shown here, indicate that if one is going to conceptualize the association by a threshold value, then ≥ 5 μg/dL captures much more of the variation in these data than does the CDC blood lead action level of ≥ 10 μg/dL.

The third model enters a dummy variable for each blood lead level (2, 3, 4, … 9, ≥ 10 μg/dL). The last dummy variable combines all blood lead levels ≥ 10 μg/dL, and the referent group is a blood lead level of 1 μg/dL. This scoring is the most flexible and allows a distinct estimate at each blood lead level score.

For the 4th-grade reading analysis, the coefficient on the dummy variable for a blood lead level of 2 μg/dL is negative and marginally significant at *p* = 0.05. The coefficients on the dummy variables for blood lead levels of 3–8 and 10 μg/dL are consistently negative and statistically significant, and generally increase in absolute magnitude as the blood lead levels increase (all *p* < 0.0001). The coefficient on the dummy variable for a blood lead level of 9 μg/dL is also negative but significant only at the *p* = 0.02 level, likely due to the small sample size in this grouping. The results for the 4th-grade mathematics analysis follow a very similar pattern to those of the reading analysis, although the coefficient on the dummy variable for a blood lead level of 2 μg/dL is significant at the *p* = 0.03 level, and the coefficient on the dummy variable for a blood lead level of 9 μg/dL is significant at the *p* < 0.0001 level.

Model 3 results demonstrate a strong dose–response effect between early childhood lead exposure and performance on elementary school achievement tests. These results indicate clearly that early childhood lead exposure has a statistically significant and negative impact on school performance at levels well below the current CDC blood lead action level. These results are consistent with the observed association between blood lead levels and elementary school achievement scores demonstrated in both the descriptive analysis and regression models 1–2. All three models indicate, net of a set of control variables, that higher blood lead levels are associated with lower test scores. The least constrained model (model 3) reveals a general decline in test scores with rising blood lead levels. Model 1 constrains this decline to be uniform across all blood lead levels. With our data, we cannot reject the latter in favor of the former; any divergence from a linear decline could be attributed to sampling variability. Model 2 can be aligned with the following question: Once we take account of high blood lead levels (i.e., ≥ 10 μg/dL) is additional variation in blood lead levels important? Results clearly indicate that blood lead levels of 5–10 μg/dL are consequential for test scores. We conclude from these various representations that early childhood blood lead levels reduce test scores and that this effect is clear even at levels < 10 and even < 5 μg/dL.

**Figure 5:**
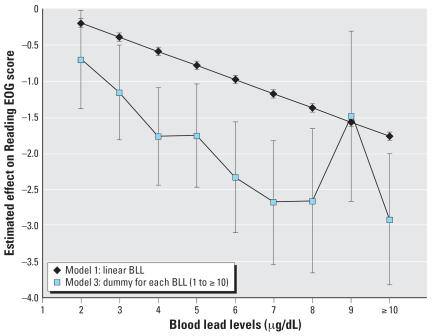
Comparing model results for 4th-grade Reading score. Based on a referent individual who was screened at 2 years of age and is a white female, living in Wake County, parents with a high school education, not enrolled in the school lunch program, and who does not use a computer every day. Baseline score is 257.1.

**Figure 6:**
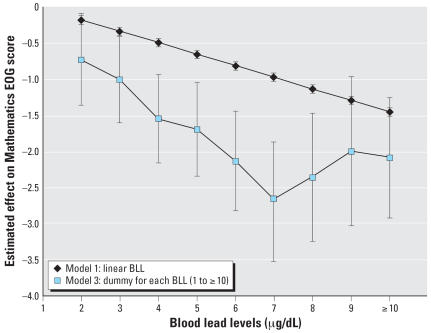
Comparing model results for 4th-grade Mathematics scores. Based on a referent individual who was screened at 2 years of age and is a white female, living in Wake County, parents with a high school education, not enrolled in the school lunch program, and who does not use a computer every day. Baseline score is 262.6.

Given the statistical measures of model fit provided in Tables 2 and 3 (adjusted *R*^2^, AIC, and root MSE), all three models show adequate and substantially similar model fit. Figures 5 and 6 graphically summarize the results of models 1 and 3 for the 4th-grade reading and mathematics analyses graphically. These figures aptly demonstrate that test scores decline as early childhood blood lead levels increase. Because model 3 allows a distinct estimate at each blood lead level score, it is useful to compare it directly with model 1, which constrains the effect of a one-unit increase in blood lead level to be uniform across observed scores. Figures 5 and 6 show that the decline in both reading and mathematics scores is steeper at lower blood lead levels than at higher blood lead levels.

## Conclusions

As perhaps is best seen in Figures 5 and 6, using a variety of modeling approaches, blood lead levels in early childhood are related to educational achievement in early elementary school as measured by performance on end-of-grade testing. According to 2003–2004 NHANES data, 50% of children 1–5 years old nationwide are estimated to have blood lead levels of ≥ 3 μg/dL (National Center for Health Statistics 2006). Thus as many as half the children in the United States are experiencing negative effects associated with lead exposure—a significantly higher proportion than the 2.3% estimated using the CDC’s current blood lead action level of 10 μg/dL.

In addition, early childhood lead exposures appear to have more impact on performance on the reading than on the mathematics portions of the EOG, although the differences may not be statistically significant. This differential impact on reading versus mathematics is consistent with previous studies (Fulton et al. 1987; Lanphear et al. 2000).

The estimated effects are mean effects—averages of the adverse effects across children. These shifts will affect a substantial number of children at any given test threshold. For example, at the low end of the distribution, the impact of lead on EOG test results is sufficient to ensure that some students, who would otherwise have passed the test, will fail. This in turn has implications for retention in grade. In addition, at the high end of the distribution, the impact of lead on EOG test results will essentially block some students from gaining access to the enriched resources provided through advanced and intellectually gifted (AIG) programs. As is true for many states, the use of EOG scores to determine placement into AIG programs is ubiquitous in North Carolina. These two phenomena are especially troubling given that we know that low-income and minority children are systematically exposed to more lead in North Carolina and nationally.

It is also notable that the size of the coefficients on the lead variables is very meaningful compared with other covariates that we typically think of as profoundly influential on educational outcomes. For example, in model 3, in the 4th-grade reading analysis, a blood lead level of 3 μg/dL has an impact roughly equal to 59% of the impact of participating in the free or reduced-price lunch program (the classic poverty indicator in school data). A blood lead level of 4 μg/dL has an impact roughly equal to 90% of the impact of participating in the free or reduced-price lunch program, and a blood lead level of ≥ 6 μg/dL has a greater impact. In addition, the size of the coefficients, which may seem small compared with the constants (~ 250–265), are in fact quite substantial in context. For example, across North Carolina in 2003–2004, the interquartile range for 4th-grade reading EOG test scores spanned 12 points, and the interquartile range for 4th-grade mathematics EOG test scores spanned 10 points. Thus a blood lead level of 5 μg/dL is associated with a decline in EOG reading (mathematics) scores that is roughly equal to 15% (14%) of the interquartile range.

This study has several limitations. First, previous cohort studies have shown that direct measures of parental IQ and quality of the home environment are important explanators of test performance in children (Bacharach and Baumeister 1998). Our study was limited in that we could incorporate only indirect measures of parental IQ via parental education [see Neisser et al. (1996) for a justification of this proxy] and poverty measures (free or reduced-price lunch program and charter school) to substitute for quality of the home environment. To the extent that lead exposure may be correlated with parental IQ or the home environment, by relying on these proxies we may be overestimating the effects of early childhood lead exposure on end-of-grade test performance. Our study does, however, rely on a substantially larger sample size than many previous studies. Second, the children screened for lead are not randomly drawn from the population, raising concerns of selectivity bias. We are in the process of obtaining the data that would allow us to diagnose and directly address any issues of selectivity bias.

Despite its limitations, this study enriches the existing literature on the link between early childhood lead exposure and cognitive outcomes. Our emphasis on a population-level analysis of children who are roughly the same age linked to previous (rather than contemporaneous) blood lead levels using achievement (rather than aptitude) outcome complements the important work in this area by previous researchers (Canfield et al. 2003; Fulton et al. 1987; Lanphear et al. 2000, 2005; Schwartz 1994). Our results suggest that the relationship between lead levels and cognitive outcomes are robust across outcome measures and at low levels of lead exposure.

In conducting this analysis, we noted that a higher proportion of black children had higher blood lead levels. Thus, in future analyses we plan to explore whether this differential exposure to lead in early childhood might explain part of the so-called achievement gap. We are also interested in following the same children through their elementary, middle school, and high school years to assess the persistence of the effects we note here.

## Figures and Tables

**Table 1:** Arithmetic mean blood lead levels (BLL; μg/dL) among 4th-grade children whose screening data linked with education data.

	Reading data set		Mathematics data set	
Variable	Mean BLL	Sample size	Mean BLL	Sample size
Race				
White	3.71	3,853	3.70	3,861
Black	5.19	4,750	5.19	4,766
Household income				
Not enrolled in free/reduced-price lunch program	3.91	5,194	3.90	5,201
Enrolled in free/reduced-price lunch program	5.47	3,409	5.47	3,426
Parental education				
Completed graduate school	3.57	244	3.57	245
Completed college	3.61	1,309	3.60	1,312
Some post–high school education	4.03	2,779	4.04	2,780
Completed high school	4.99	3,572	4.99	3,584
Some high school education	6.19	699	6.16	706
Overall	4.52	8,603	4.53	8,627

**Table 2:** Results of multivariate regression models for 4th-grade Reading EOG score data (*n* = 8,603).

	Model 1: linear BLL		Model 2: dummy of BLL5–9 and dummy of BLL ≥10		Model 3: dummy for each BLL	
Response variable	Coefficient	*p* > *t*	Coefficient	*p* > *t*	Coefficient	*p* > *t*
BLL (continuous, linear term)	−0.20	0.00				
Dummy for BLL 5–9 μg/dL			−1.01	0.00		
Dummy for BLL = 2 μg/dL					−0.70	0.05
Dummy for BLL = 3 μg/dL					−1.16	0.00
Dummy for BLL = 4 μg/dL					−1.77	0.00
Dummy for BLL = 5 μg/dL					−1.75	0.00
Dummy for BLL = 6 μg/dL					−2.33	0.00
Dummy for BLL = 7 μg/dL					−2.68	0.00
Dummy for BLL = 8 μg/dL					−2.66	0.00
Dummy for BLL = 9 μg/dL					−1.49	0.02
Dummy for BLL ge;10 μg/dL			−1.75	0.00	−2.92	0.00
Male (1 for male; 0 for female)	−1.43	0.00	−1.44	0.00	−1.41	0.00
Black (1 for black; 0 for white)	−4.59	0.00	−4.58	0.00	−4.46	0.00
Uses computer every day at home	−2.26	0.00	−2.24	0.00	−2.20	0.00
Enrolled in free or reduced-price lunch program	−2.03	0.00	−2.04	0.00	−1.97	0.00
Parents with some high school education	−2.18	0.00	−2.21	0.00	−2.17	0.00
Parents with some post–high school education	2.74	0.00	2.75	0.00	2.71	0.00
Parents completed college	4.73	0.00	4.74	0.00	4.67	0.00
Parents completed graduate school	7.49	0.00	7.51	0.00	7.45	0.00
Age when child was screened for lead	−0.81	0.00	−0.81	0.00	−0.83	0.00
Charter school	−3.66	0.00	−3.68	0.00	−3.65	0.00
Alamance–Burlington school system	−1.34	0.00	−1.36	0.00	−1.35	0.00
Chatham County school system	−1.74	0.00	−1.76	0.00	−1.66	0.00
Durham County school system	−1.23	0.00	−1.26	0.00	−1.26	0.00
Granville County school system	−1.69	0.00	−1.74	0.00	−1.66	0.00
Chapel Hill–Carrboro school system	1.26	0.03	1.17	0.04	1.13	0.05
Orange County school system	−0.76	0.07	−0.76	0.07	−0.71	0.09
Person County school system	0.67	0.08	0.63	0.10	0.74	0.05
Constant	258.09	0.00	257.67	0.00	258.74	0.00
Adjusted *R*^2^	0.34		0.34		0.34	
AIC	6.81		6.81		6.81	
Root MSE	7.29		7.29		7.28	

BLL, blood lead level. The mean 4th-grade Reading EOG score for this sample is 251.4, the median 252, and the SD 9.0. The interquartile range was 13.

**Table 3:** Results of multivariate regression models for 4th-grade Mathematics EOG score data (*n* = 8,627).

	Model 1: linear BLL		Model 2: dummy of BLL5-9 and dummy of BLL ≥ 10		Model 3: dummy for each BLL	
Response variable	Coefficient	*p* > *t*	Coefficient	*p* > *t*	Coefficient	*p* > *t*
BLL (continuous, linear term)	−0.16	0.00				
Dummy for BLL 5–9 μg/dL			−1.05	0.00		
Dummy for BLL = 2 μg/dL					−0.71	0.03
Dummy for BLL = 3 μg/dL					−0.99	0.00
Dummy for BLL = 4 μg/dL					−1.53	0.00
Dummy for BLL = 5 μg/dL					−1.68	0.00
Dummy for BLL = 6 μg/dL					−2.13	0.00
Dummy for BLL = 7 μg/dL					−2.64	0.00
Dummy for BLL = 8 μg/dL					−2.35	0.00
Dummy for BLL = 9 μg/dL					−1.99	0.00
Dummy for BLL ≥ 10 μg/dL			−1.03	0.00	−2.07	0.00
Male (1 for male; 0 for female)	0.10	0.46	0.09	0.51	0.12	0.41
Black (1 for black; 0 for white)	−4.53	0.00	−4.50	0.00	−4.40	0.00
Uses computer every day at home	−1.80	0.00	−1.78	0.00	−1.74	0.00
Enrolled in free or reduced-price lunch program	−1.57	0.00	−1.57	0.00	−1.51	0.00
Parents with some high school education	−1.85	0.00	−1.87	0.00	−1.83	0.00
Parents with some post–high school education	2.38	0.00	2.39	0.00	2.35	0.00
Parents completed college	3.93	0.00	3.92	0.00	3.86	0.00
Parents completed graduate school	6.50	0.00	6.52	0.00	6.46	0.00
Age when child was screened for lead	−0.87	0.00	−0.88	0.00	−0.90	0.00
Charter school	−4.35	0.00	−4.37	0.00	−4.33	0.00
Alamance–Burlington school system	−0.49	0.06	−0.51	0.05	−0.51	0.05
Chatham County school system	−2.40	0.00	−2.41	0.00	−2.32	0.00
Durham County school system	−1.10	0.00	−1.14	0.00	−1.15	0.00
Granville County school system	−1.84	0.00	−1.89	0.00	−1.82	0.00
Chapel Hill–Carrboro school system	−1.11	0.04	−1.20	0.03	−1.23	0.02
Orange County school system	−0.65	0.07	−0.65	0.07	−0.61	0.09
Person County school system	0.29	0.40	0.25	0.48	0.34	0.33
Constant	263.70	0.00	263.42	0.00	264.37	0.00
Adjusted *R*^2^	0.35		0.35		0.35	
AIC	6.57		6.57		6.57	
Root MSE	6.47		6.47		6.45	

BLL, blood lead level. The mean 4th-grade Reading EOG score for this sample is 257.8, the median 258, and the SD 8.0. The interquartile range was 11.
